# Type B Lactic Acidosis in a Patient with Mantle Cell Lymphoma

**DOI:** 10.1155/2023/7021123

**Published:** 2023-08-16

**Authors:** Ikemsinachi C. Nzenwa, Margaret Berquist, Toby J. Brenner, Aida Ansari, Hamid D. Al-Fadhl, Michael Aboukhaled, Shivani S. Patel, Ethan E. Peck, Mahmoud D. Al-Fadhl, Anthony V. Thomas, Nuha Zackariya, Mark M. Walsh, Jose A. Bufill

**Affiliations:** ^1^Saint Joseph Regional Medical Center, Mishawaka, Indiana, USA; ^2^Indiana University School of Medicine South Bend Campus, Notre Dame, Indiana, USA; ^3^Michiana Hematology-Oncology, Mishawaka, Indiana, USA

## Abstract

Type B lactic acidosis is an uncommon medical emergency in which acid production overwhelms hepatic clearance. This specific etiology of lactic acidosis occurs without organ hypoperfusion and has been most commonly described in patients with hematologic malignancies but also in patients with solid tumors. The mechanism by which cancer cells switch their glucose metabolism toward increasingly anaerobic glycolytic phenotypes has been described as the “Warburg effect.” Without treating the underlying malignancy, the prognosis for patients diagnosed with malignancy-related type B lactic acidosis is extremely poor. Here, we present a case of a 66-year-old male who was diagnosed with type B lactic acidosis secondary to mantle cell lymphoma. Bicarbonate drip was started to correct the lactic acidosis. The patient was also immediately treated with rituximab chemotherapy combined with rasburicase to avoid the hyperuricemia associated with tumor lysis syndrome. He responded to the early treatment and was discharged with normal renal function. Type B lactic acidosis secondary to hematologic malignancy is important to recognize. In order to successfully treat this syndrome, early diagnosis and simultaneous treatment of the imbalance of lactic acid levels and the underlying malignancy are necessary.

## 1. Introduction

Lactic acidosis is a medical emergency that can result in severe hemodynamic consequences. The most common etiology is type A lactic acidosis, which often reflects tissue hypoxia due to hypoperfusion [1–3]. A minority of cases are classified as type B lactic acidosis, which often occurs without organ hypoperfusion and has been described in association with hematologic and solid malignancies [4–8]. Type B lactic acidosis may also result from interference of cellular metabolism or nutritional deficiency [4–6, 9].

**Figure 1:**
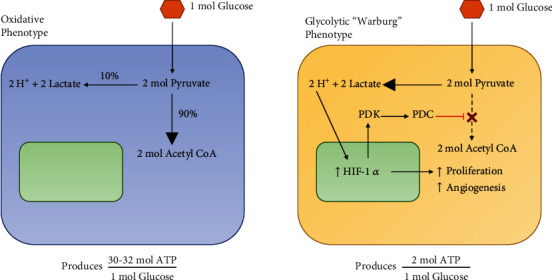
Normal oxidative pathway vs. the Warburg effect. The normal oxidative pathway used by healthy cells is oxygen-dependent and efficiently converts glucose into adenosine triphosphate. The malignant phenotype as explained through the Warburg effect is independent of oxygen concentration and inefficiently converts glucose into adenosine triphosphate.

In patients with type B lactic acidosis secondary to malignancy, the preponderance of lactate is produced within tumor cells under the influence of the metabolic derangement known as the “Warburg effect.” The Warburg effect refers to the switching of cancer cells' primary glucose metabolism from the oxidative pathway to the anaerobic glycolytic pathway, resulting in lactate buildup and hypoglycemia [10]. While the full mechanism behind the Warburg effect is outside the scope of this report, the glycolytic shift toward the generation of lactate may be partly explained by enhanced glycolytic activity in the cancerous cells following oncogenic lesions overexpressing hypoxia-inducible factor-1*α* (Figure 1) [11–13].

Mantle cell lymphoma (MCL) is a rare and aggressive form of B-cell non-Hodgkin's lymphoma. It is rarely associated with type B lactic acidosis via the Warburg effect [14]. Here, we present a case of successfully treated type B lactic acidosis in a patient with recently diagnosed MCL. No written consent was obtained from the patient as there is no patient identifiable data included in this case report.

## 2. Case Presentation

**Figure 2:**
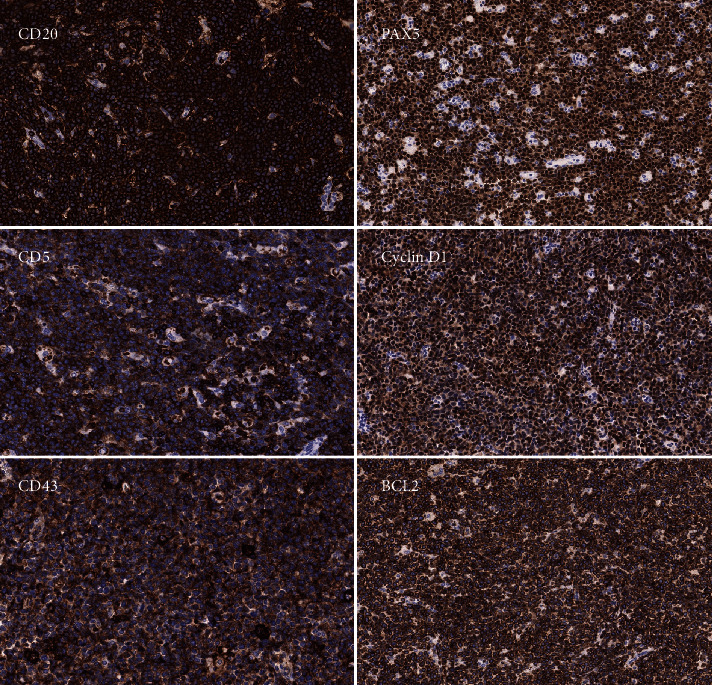
Immunohistochemical studies of right axillary lymph node neoplastic cells were performed and were positive for CD20, PAX5, CD5, cyclin D1, CD43, and BCL2 which are commonly found in MCL [15–17].

A 66-year-old male with history of diabetes mellitus, cardioembolic stroke, and patent foramen ovale was diagnosed with MCL two weeks prior to presentation. Diagnosis of MCL was confirmed by immunohistochemical studies of neoplastic cells from right axillary lymph nodes that were positive for CD20, PAX5, CD5, cyclin D1, CD43, and BCL2 tumor markers associated with MCL (Figure 2). He had been gradually declining with decreased appetite, worsening abdominal distension and bloating, and significant lymphadenopathy for the past week. He presented to the emergency department before initiation of chemotherapy treatment. On arrival, the patient was mildly tachycardic and tachypneic, but the rest of his vitals were normal. On exam, he appeared frail and older than stated age. His abdomen was rotund and distended, and he had pitting edema bilaterally. He was found to have a 2/6 systolic ejection murmur. The patient had acute kidney disease with metabolic derangements characterized by elevated blood urea nitrogen (BUN) (57 mg/dL) and creatinine (2.66 mg/dL). Labs also demonstrated low serum bicarbonate (16 mEq/L) and elevated lactic acid level (3.8 mMol/L), revealing lactic acidosis. The patient was admitted to the intensive care unit for lactic acidosis, dehydration, and kidney disease. He was hydrated aggressively and started on a bicarbonate drip to treat lactic acidosis.

On the second day of admission, the patient's lactic acid level increased to 7.0 mMol/L, and he was noted to have abdominal pain with a positive *Clostridium difficile* toxin specimen of the stool. A computed tomography scan of the abdomen and pelvis was done revealing extensive retroperitoneal and intra-abdominal lymphadenopathy with splenomegaly. There was no evidence of bowel ischemia or hydronephrosis that could be contributing to the lactic acidosis. After multiple consultations, a diagnosis of type B lactic acidosis secondary to MCL was made. He was given a fluid challenge as well as intravenous rituximab with rasburicase to prevent acute urate nephropathy due to his chronic renal insufficiency.

By the fourth day of admission, the patient's clinical condition started to improve, and he was transitioned out of the intensive care unit. His bicarbonate levels improved, so the bicarbonate drip was terminated, and he was started on normal saline. He continued to respond to therapy with normalization of creatinine (1.13 mg/dL), BUN (19 mg/dL), and serum lactic acid levels. Supportive care was continued for ongoing volume overload and acute kidney injury, and he was discharged two weeks after the diagnosis of type B lactic acidosis.

## 3. Discussion

The “Warburg effect,” which was initially described in 1923, provides the most complete explanation of the hypothesized mechanism behind type B lactic acidosis in malignancies and is an increasingly recognized hallmark of cancer cells [18]. Cancer cells upregulate glucose transporters and hexokinases, but as they grow, these cells outpace their blood supply and create a microenvironment with fluctuating concentrations of oxygen, contributing to genomic instability, tumor growth, metastasis, and angiogenesis [5, 11, 19–22]. Environmental pressure selects clones that survive via an upregulated glycolytic phenotype, resulting in inefficient production of adenosine triphosphate, excessive lactic acid and proton production and accumulation, and acidosis (Figure 1) [11, 19–21, 23–25].

Type B lactic acidosis has been described in conjunction with various hematologic malignancies and occasionally in cases involving solid tumors [5–7, 26]. Presentation in solid tumors is uncommon, as Liu et al. noted that between 2010 and 2020, there have only been 15 case reports regarding type B lactic acidosis with solid tumor malignancy. Of those cases, only two patients achieved long-term survival, with a large fraction of fatalities occurring within days of the diagnosis [27]. With lymphomas, the prognosis is slightly better [5–7, 26].

Hematological malignancies, which inherently impact both the circulatory and lymphatic systems, present serious challenges to physicians attempting to treat the systemic consequences of the disease. Type B lactic acidosis has been reported with hematologic malignancies such as acute and chronic leukemias, histiocytosis, and Hodgkin's lymphoma. A literature review regarding type B lactic acidosis secondary to malignancy demonstrated that type B lactic acidosis served as a poor prognostic marker for patients and that most cases were fatal despite intense intervention [28]. Previous cases treated acidemia with intravenous bicarbonate infusion, thiamine replacement therapy, hemodialysis, or chemotherapy [29–32]. In a literature review of type B lactic acidosis secondary to lymphomas, only seven patients survived after initiation of chemotherapy with normalization of their lactic acid out of 29 cases reported [29].

There are very few reported cases of MCL associated with type B lactic acidosis [14, 33–36]. We have described a case of successfully treated type B lactic acidosis in a patient with MCL. Immediately, the patient was treated with rituximab chemotherapy combined with rasburicase in order to avoid the hyperuricemia associated with tumor lysis syndrome. While waiting for the effect of chemotherapy, the patient was vigorously resuscitated with bicarbonate infusions. Within days, the patient responded to treatment and was discharged with improvement of renal function. Of the cases of MCL associated with type B lactic acidosis reported in the literature, four patients survived and one expired (Table 1). Each patient was treated with chemotherapy targeting the MCL and with sodium bicarbonate targeting the lactic acidosis. A common thread amongst the patients who survived, including our patient, is that each patient was recently diagnosed with MCL at the time of presentation. MCL is highly responsive to chemotherapy with initial treatments; however, it has frequent relapse patterns with poor long-term outcomes, rendering therapy less effective with time [37, 38]. This suggests that the most effective treatment for patients with type B lactic acidosis secondary to newly diagnosed MCL should be to address the underlying malignancy itself rather than solely treating lactic acidosis. Thus, it is possible to successfully treat type B lactic acidosis if it manifests in the early stages of MCL with a combined regimen of chemotherapy and sodium bicarbonate.

## 4. Conclusion

When treating patients with hematological malignancies that present with idiopathic degeneration of respiratory status, type B lactic acidosis should be considered as a possible diagnosis. Metformin-associated lactic acidosis is a much more common form of significantly elevated lactic acidosis which often responds to aggressive treatment with intravenous fluids, bicarbonate, and renal replacement therapy when indicated [39–41]. In order to successfully treat a type B lactic acidosis emergency related to a rapidly growing neoplasm such as MCL, directing clinical action at both the imbalance of lactic acid levels and the underlying malignancy simultaneously is necessary. Rebalancing lactic acid production and clearance through renal replacement therapy, thiamine replacement therapy, and buffering the acid with bicarbonate infusion, as well as aggresively treating the underlying malignancy through chemotherapy, may be the most successful strategy.

## Figures and Tables

**Table 1:** Previous similar cases.

Case	Age	Sex	Diagnosis	Therapy	Outcome
Ohtsubo et al. [33]	77	Male	Recently diagnosed blastoid variant of MCL, lactic acidosis	THP-COP^∗^, EPOCH^†^, rituximab	Survived
Diab et al. [34]	81	Male	Recently diagnosed MCL, lactic acidosis, hypoglycemia	Rituximab, cyclophosphamide, vincristine, prednisone	Survived
Wahab et al. [35]	67	Male	Recently diagnosed blastoid variant of MCL, lactic acidosis, hyperuricemia	Rasburicase, oral allopurinol, dexamethasone, VR-CAP^‡^	Survived
Dejman and Riveros [36]	66	Male	Stage IV MCL, lactic acidosis, bladder cancer	Bendamustine, rituximab, continuous venovenous hemofiltration, IV thiamine, chemotherapy	Expired
Chan et al. [14]	59	Male	Recently diagnosed MCL, lactic acidosis	R-CHOP^§^	Survived

^∗^Tetrahydropyranyl doxorubicin, cyclophosphamide, vincristine, and prednisolone. ^†^Etoposide, prednisone, vincristine, and doxorubicin. ^‡^Bortezomib, rituximab, cyclophosphamide, doxorubicin, and prednisone. ^§^Rituximab, cyclophosphamide, doxorubicin, vincristine, and prednisone. All patients received sodium bicarbonate as part of their therapies.

## Data Availability

No data were used to support the findings of this study.
